# *Leptadenia reticulata* (Retz.) Wight & Arn. (Jivanti): Botanical, Agronomical, Phytochemical, Pharmacological, and Biotechnological Aspects

**DOI:** 10.3390/molecules22061019

**Published:** 2017-06-19

**Authors:** Sudipta Kumar Mohanty, Mallappa Kumara Swamy, Uma Rani Sinniah, Maniyam Anuradha

**Affiliations:** 1 Instituto de Biología, Universidad Nacional Autónoma de México, Ciudad Universitaria, Coyoacan, Ciudad de México C.P. 04510, Mexico; su_sudeepta@yahoo.co.in; 2 School of Life Sciences, Padmashree Institute of Management and Sciences, Kommagatta, Kengeri, Bengaluru 560060, India; pimsprincipal@gmail.com; 3 Department of Crop Science, Faculty of Agriculture, Universiti Putra Malaysia, Serdang, Selangor, Darul Ehsan 43400, Malaysia

**Keywords:** Jivanti, *Leptadenia reticulata*, traditional medicine, herb, therapy, rasayana, galactagogue, pharmacology, biological activities, medicinal plant

## Abstract

*Leptadenia reticulata* (Retz.) Wight & Arn. (Apocynaceae), is a traditional medicinal plant species widely used to treat various ailments such as tuberculosis, hematopoiesis, emaciation, cough, dyspnea, fever, burning sensation, night blindness, cancer, and dysentery. In Ayurveda, it is known for its revitalizing, rejuvenating, and lactogenic properties. This plant is one of the major ingredients in many commercial herbal formulations, including Speman, Envirocare, Calshakti, Antisept, and Chyawanprash. The therapeutic potential of this herb is because of the presence of diverse bioactive compounds such as α-amyrin, β-amyrin, ferulic acid, luteolin, diosmetin, rutin, β-sitosterol, stigmasterol, hentricontanol, a triterpene alcohol simiarenol, apigenin, reticulin, deniculatin, and leptaculatin. However, most biological studies on *L. reticulata* are restricted to crude extracts, and many biologically active compounds are yet to be identified in order to base the traditional uses of *L. reticulata* on evidence-based data. At present, *L. reticulata* is a threatened endangered plant because of overexploitation, unscientific harvesting, and habitat loss. The increased demand from pharmaceutical, nutraceutical, and veterinary industries has prompted its large-scale propagation. However, its commercial cultivation is hampered because of the non-availability of genuine planting material and the lack of knowledge about its agronomical practices. In this regard, micropropagation techniques will be useful to obtain true-to-type *L. reticulata* planting materials from an elite germplasm to meet the current demand. Adopting other biotechnological approaches such as synthetic seed technology, cryopreservation, cell culture, and genetic transformation can help conservation as well as increased metabolite production from *L. reticulata.* The present review summarizes scientific information on the botanical, agronomical, phytochemical, pharmacological, and biotechnological aspects of *L. reticulata*. This comprehensive information will certainly allow better utilization of this industrially important herb towards the discovery of lead drug molecules.

## 1. Introduction

The utilization of plants for their therapeutic value has been known to mankind from times immemorial and has played an essential role in the various traditional systems of medicine including Ayurveda, Homeopathy, Siddha, Unani, Naturopathy, and Chinese, Tibetan, and Native American medicine [1,2,3,4]. As per the estimation of the World Health Organization, 80% of the world population is currently using herbal medicines for primary health care needs [1,3,5]. At present, drug discovery research is mainly focused on natural plant resources and their compounds. Most of the currently available therapeutically active drugs are discovered based on the knowledge available from various traditional disease treatment practices [1,2,3,6,7]. Awareness, health consciousness, thoughts on prevention being better than cure, and natural ways of healthy living has further propelled the use of herbal products. The exponential growth of nutraceutical and cosmeceutical consumers has increased the demand for plant raw materials [8,9,10]. Ayurveda has a science called Rasayana, which deals with the improvement of general health, vigor, and vitality. Among various herbs used in Rasayana, *Leptadenia reticulata* (Jivanti) has a unique place in lieu of its revitalizing, rejuvenating, and lactogenic properties [11]. *L. reticulata* is used for treating various ailments such as hematopoiesis, emaciation, cough, dyspnea, fever, burning sensation, night blindness, and dysentery [11,12]. This plant is used as one of the ingredients in many herbal formulations. The therapeutic potential of this herb is because of the presence of diverse bioactive compounds such as α-amyrin, β-amyrin, ferulic acid, luteolin, diosmetin, rutin, β-sitosterol, stigmasterol, hentricontanol, a triterpene alcohol simiarenol, apigenin, reticulin, deniculatin, and leptaculatin [12,13,14,15]. The wild resources are unable to meet the current demand because of restricted distribution and seasonal availability. Hence, cultivation remains the only sustainable alternative. Further, wild resources of *L. reticulata* have been depleted by overexploitation through various anti-social practices, and thus, it has been listed as an endangered species [16]. Although farmers and industries are ready to cultivate the herb, its low percentage of germination, non-availability of genuine plant materials, and a lack of knowledge about its cultivation practices pose a challenge for its commercial cultivation [17,18]. However, the higher market value and global demands for *L. reticulata* have forced farmers to consider cultivating it in recent times. Today, various medicinal plants are commercially cultivated to meet the ever increasing global demand for plant metabolites used by the pharmaceutical industries [19]. Nevertheless, various crop improvement strategies are yet to be adopted to develop superior varieties of medicinal plants. The use of micropropagation approach will certainly help in the large-scale production of elite genetically and chemically uniform planting material [18,19]. In spite of intensive conventional research effort and vast agronomic practices, the desired *L. reticulata* production target has not been achieved thus far. *L. reticulata* is often adulterated with many other herbs, including *Holostemma ada-kodien*, *Dendrobium ovatum*, *D. macraei*, *Flickingeria macraei*, *Cimicifuga foetida*, and *Ichnocarpus frutescens* [20,21,22,23]. Thus, the authenticity of *L. reticulata* is another great challenge in the herbal medicine industry. The commercial cultivation of any herb requires a thorough understanding of its botanical, chemical, geographical, growth, and developmental characteristics. Further, better management of plant nutrition, diseases, and pest problems will boost the commercial yield of a crop. The present review was undertaken to compile the available scientific data on the botanical, agronomical, phytochemical, pharmacological, and biotechnological aspects of *L. reticulata*. This data will certainly enable researchers to explore novel drug molecules from this multi-utilitarian herb. This comprehensive review includes scientific information retrieved from various search engines such as Google Scholar, Scopus, PubMed, and ScienceDirect.

## 2. Botanical Aspects of *L. reticulata*

### 2.1. Taxonomy

The Ayurvedic herb *L. reticulata* (Jivanti) is a member of the Apocynaceae plant family. Its taxonomic position is detailed as follows:
KingdomViridiplantaePhylumStreptophytaClassMagnoliopsidaOrderGentianalesFamily*Apocynaceae*Sub-family*Asclepiadoideae*Genus*Leptadenia*Species*Leptadenia reticulata* (Retz.) Wight & Arn.

*L. reticulata* is referred by many names in Ayurveda such as Jivanti, Jivaniya, Jivapushpa, Hemavati, Jivana, Shakashreshtha, Payaswini, Maangalya, and Madhusrava. In Siddha medicine, it is known as Keerippaalai. In India, *L. reticulata* is also called by various vernacular names as listed in Table 1. The genus *Leptadenia* is comprised of four species, namely *L. pyrotechnica* (Forssk.), *L. arborea* (Forssk.), *L. hastata* (Pers.), and *L. reticulata* (Weight and Arn.) [24,25]. Among them, *L. pyrotechnica* is a desert herb with straight stems and mostly leafless, while others are twining shrubs and bear leaves. Because of its taxonomic complexity, these three species are further stated to be comprised as a single species [25,26]. Most of these *Leptadenia* species are economically valued for their therapeutic properties. Among them, *L. reticulata* is one of the most important medicinal herbs used in Ayurveda for promoting vitality and life.

### 2.2. Origin and Distribution

Although the true origin of *L. reticulata* has not been identified so far, the description in the oldest scripture of Hinduism (*Atharvaveda*) indicates that it probably originated in India. In India, it is mainly found in Rajasthan, Gujarat, Punjab, the Himalayan ranges, Khasi Hills, Sikkim, Deccan Plateau, Konkan ranges, Karnataka, and Kerala up to an altitude of 2000 m [25,27]. Apart from India, it is also reported to be distributed in the tropical and subtropical parts of Africa, Burma, Nepal, Sri Lanka, Malay Peninsula, Cambodia, the Philippines, Mauritius, and Madagascar [20,22,28]. People in Gujarat and Kathiawar use this plant as a pot herb [22]. After intensive field survey in 12 districts in Western Rajasthan (Indian Thar Desert), Panwar and Tarafdar [29] reported the occurrence of *L. reticulata* from various districts. This species was also found on hedges, in open forests, and on the lower slopes of hills [30]. Because of its high demand, it is commercially cultivated in some parts of India [20,27].

### 2.3. Morphology

**Figure 1:**
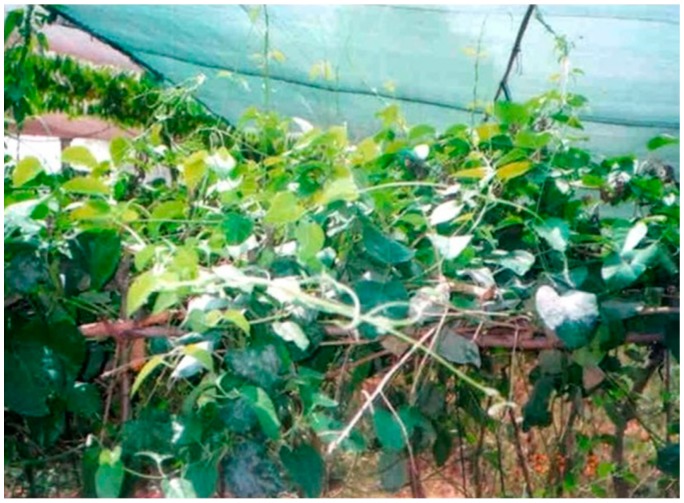
Morphological view of *L. reticulata.*

It is a perennial much branched, twining, and laticiferous climber (Figure 1). Mature stems are pale yellowish with deeply cracked barks, and younger ones are greenish glabrous. The leaves are quite big (4–7.5 cm long and 2–5 cm wide), simple, opposite, ovate or ovate-oblong (3–9 cm × 1.1 cm), cordate, and finely pubescent above [27]. The petioles are up to 2.5 cm long. The plant flowers profusely (up to 270 flowers per plant), and complete opening of flower buds takes 25–28 days. Peak anthesis is observed between 9:00 and 9:30 a.m., and flowers open for 4 to 5 days. Anther dehiscence occurs between 11:00 a.m. and 1:00 p.m. on the fourth day during which the flowers are nearly at the wilting stage [30]. Flowering occurs between July and October and fruiting between September and December. Flower is yellowish with lateral or sub auxiliary umbellate cymes. Calyx is five-lobed; lobes are ovate, sub-acute, and silky with small hairs on the surface. Corolla is rotate and fleshy pubescent with short tube. Staminal column is short. Corona is five-lobed, gamopetalous, spreading with spur from the interior of each lobe. Stamens are five and adnate to the base of the corolla tube; filaments fuse with the stigmatic head to form a five-angled disc called gynostegium. Anthers are without membranous appendages. Pollen grains are arranged on the lateral side of stigma. Ovary is bicarpellary with marginal placentation. Fruit is follicular, sub woody, turgid, approximately 6.3–9 cm long, tapering, and green [11,25]. Fruits mature in 102–158 days and contain up to 448 seeds. Seeds are ovate oblong, tapering about 6 mm in diameter. Presently, there are no certified varieties available [30]. However, based on leaf morphology, this plant is mainly categorized into two variants i.e., broad-leaved plants and narrow-leaved plants. The broad-leaved germplasm was evaluated to be predominant than narrow one and yields higher amount of roots and other phytochemicals. The roots are rough and white in color with longitudinal ridges and furrows. Roots are cylindrical and twisted irregularly with longitudinal ridges. Root length varies up to 1 m or more. Stem is yellowish white in color with longitudinal lenticels [25]. Mammen et al. [22] reported that the lower epidermis of the leaf contain anisocytic stomata, and the presence of multicellular, uniseriate, and smooth trichomes are the striking features offor identifying adulteration in *L. reticulata*. The epidermal layer of the leaf is composed of rectangular cells, and mesophyll consists of 3–4 layers of palisade and spongy parenchymal layers [31]. Arc-shaped vascular bundles with lignified xylem and nonlignified phloem were observed. The cross section of the stem consists of single layer of elongated epithelial cells having trichomes. The cortex below the epidermis contains thin-walled parenchymatous cells. The cambium produces secondary xylem and phloem producing continuous ring wood [31]. Phelloderm contains scattered stone cells. Lignified stone cells found in the outer phloem, intraxylary phloem, and non-articulated laticifers are the microscopic distinguishing characters of the stem of *L. reticulata.*

## 3. Agronomical Aspects of *L. reticulata*

### 3.1. Climate and Soil

The plant grows well in tropical and subtropical climate and requires moderate rainfall and relative humidity. This plant also grows in arid regions, which are characterized by sandy soil, low organic matter, and rainfall deficit. Black soil is found to be good for cultivation; however, red laterite soil is also suitable for its satisfactory growth. Open sunlight and support is necessary for healthy and vigorous growth.

### 3.2. Propagation Technique

Plants reproduce vegetatively from stem cuttings, roots, and vines. Evaluation of various planting materials such as stem cuttings, root cuttings, and vine cuttings revealed that among all parts propagation using healthy and strong stem cuttings is the most successful. Maintenance of high humidity around the cutting was found to be a critical factor to reduce the evaporative loss of water from cuttings. High humidity was maintained by covering the planting material with clear plastic bags. After rooting, the plastic bag can be removed. Treating the cuttings with root-promoting compounds is found to be a valuable tool in stimulating root formation. Newly rooted cuttings should not be planted directly. The plants can instead be transplanted into a container or a bed before transferring them to a permanent location to increase the chances of survival. Although the fruits contain fairly large quantity of seeds, the number of seedlings was less because of low germination rate and limited availability. Fruits turn ripe during November to December. Seeds are collected before the fruits dehisce, and they are dried and stored. After soaking in water for 4–5 h, the seeds are sown on nursery bed with thick layer of sand. About 1–1.5-month-old seedlings were transferred to the main field [30]. As per the agronomic study carried out by the Department of Horticulture, University of Agricultural Science, Bengaluru; Dhanvanthari Vana, Department of Forestry, Government of Karnataka; Bengaluru University, Bengaluru; State Department of Horticulture, Hulimavu; and Biotechnology Centre, Bannerghatta Road, Bengaluru, the period of February-March is suitable for planting the cuttings [30]. The cuttings of 12–15 cm long with 3–4 nodes were treated in antifungal agent and root-inducing hormones to get better rooting response. Rooted cuttings were transferred to polybags filled with Farm Yard Manure (FYM) and red earth in the ratio of 1:1 after 45 days. Three-month-old saplings intact in the soil were transferred to the plot prepared in the main field.

### 3.3. Spacing of Propagules

Intensive study was conducted at field level for yield and biomass by planting at different levels of spacing. Sapling survival rate at a spacing of 2 m × 1 m of pit size 45 cm^3^ and nearly 5000 plants planted per hectare was found to be ideal for maximum yield [30,32].

### 3.4. Preparation of Land and Fertilizer

The lands were ploughed three to four times and the soil was made finer. The plot of convenient size was prepared with good irrigation facility. Pits of 45 cm^3^ filled with FYM and top soil in the ratio of 1:1 with a spacing of 2 m × 1 m is ideal for plantation [20,30]. The months of February and March are more favorable for planting the cuttings [30]. It is also suggested that the pit should be dug deeply to facilitate the growth of the root. Cuttings can also be planted directly in polybags or seed pan filled with mixture of sand, FYM, and red earth in the ratio of (1:1:1) for better results. Kasera and Shukla [20] have reported the propagation of *L. reticulata* from seeds. They found that *L. reticulata* grows well in sandy loam to clay soil with pH 7.5–8.3. High content of FYM in soil was found to be more suitable for maximum growth and biomass [26,30]. Different fertilizers such as arbuscular mycorrhizae (AM) (100 g soil/plant), FYM (8–10 tons/ha; 5.77 g/plant), Hexameal (an organic manure; 40 q/ha; 2.31 g), nitrogen, phosphorus, potassium (NPK): full dose (60:40:30 kg/ha), and NPK: half dose (30:20:15 kg/ha) were evaluated for optimum growth [20,26]. Results revealed that FYM-treated plants showed better plant growth compared to NPK treated plants after four months. In another study by Nagarajaiah et al. [32], the application of NPK (100:200:200 g/vine) showed a superior increase of plant growth, number of primary branches, plant spread, leaf area, and stem girth. Likewise, the fresh plant weight (377–13,330 g per vine) was also noticed after 6, 12 and 18 months. While studying the distribution of three endangered medicinal plant species such as (*L. reticulata*, *Mitragyna parvifolia*, and *Withania coagulans*) and their association with Arbuscular Mycorrhizal Fungi (AMF), Panwar and Tarafdar [29] found that better establishment of these medicinal plants can be achieved by using AMF [29]. These fungi enhance the absorption of phosphorus (P) and other elements, improve water uptake, and enable the plants to withstand high temperatures. Similarly, the role of AMF in the establishment and conservation of endangered plant species is also reported by Panwar and Vyas [33].

### 3.5. Irrigation

As irrigation plays an important role, an adequate amount of water must be supplied for the overall growth and development of the plant. Among the different methods tested, such as sprinkler irrigation, surface irrigation, and drip or tickle irrigation, furrow irrigation twice a week for two to three months after planting in the field is preferred. Later, the irrigation may be done at an interval of 8–15 days. Drip or tickle irrigation, where water is supplied directly to the roots of the plants in small amounts, may be a good second choice [30].

### 3.6. Control of Weeds

Manual control of weeds and earthing up at regular intervals of one to two months is found to be beneficial [30].

### 3.7. Crop Protection

Control measures need to be undertaken against certain common infectious diseases and pests. Powdery mildew is reported to be a serious problem affecting the plant during the winter months. Various fungal diseases like leaf spot and leaf blight are also commonly observed. Infestation by leaf-eating caterpillars, aphids, and mites is commonly noticed which can be controlled manually or by spraying prophylactic sprays of monocrotophos (0.15%), dicofol (0.2%) etc. To protect the crop from termites, the soil mixture should be treated with phorate granules before transplanting them. The use of chlorpyrifos 20 EC in 20 mL L^−1^ water solution is beneficial to control termite attack [20]. Methyl parathion dust 20 kg ha^−1^ and nuvacron 1 mL L^−1^ are used to control the manifestation of grasshoppers in the rainy season and aphids and ladybird beetles in the winter season, respectively [20]. Leaf wilting is occasionally noticed at various stages and can be controlled by phytosanitary measures and drenching the affected vines with 0.15% carbendazim [30].

### 3.8. Intercropping

Being a climber, *L. reticulata* needs a host plant or a stalk to support its growth. Healthy growth is noticed when the plant is grown in partial shady areas than in completely open areas. Therefore, it should be preferably intercropped at the base of certain common trees for support. Hence, intercropping is recommended for this plant to achieve the benefit of economizing water cost and controlling diseases and pests [30]. Pests are less abundant when planted as intercrops than monocrops. The requirement of huge land space and physical support for climbing are other factors that increase the cost of cultivation when planted as a monocrop.

### 3.9. Harvesting

The crop can be maintained in the field for 10–15 years. The harvesting is preferably done twice a year without removing the root, which can serve as a future planting material or a root stalk. It is reported that higher yield can be obtained when harvested after 18 months [32]. At this stage, the fresh yield of dry roots and biomass was found to be the maximum. Fruiting of *L. reticulata* takes place between December and February, maturation continues until May, and dehiscence takes place between June and July. The favorable season for harvesting is between January and February when the leaves dry up. After harvesting, the roots and leaves are cut into required size and dried retaining the moisture content at 10% for storing. Six to seven hundred kilograms of dry weight root per hectare per year yield was reported [30].

## 4. Phytochemistry

**Figure 2:**
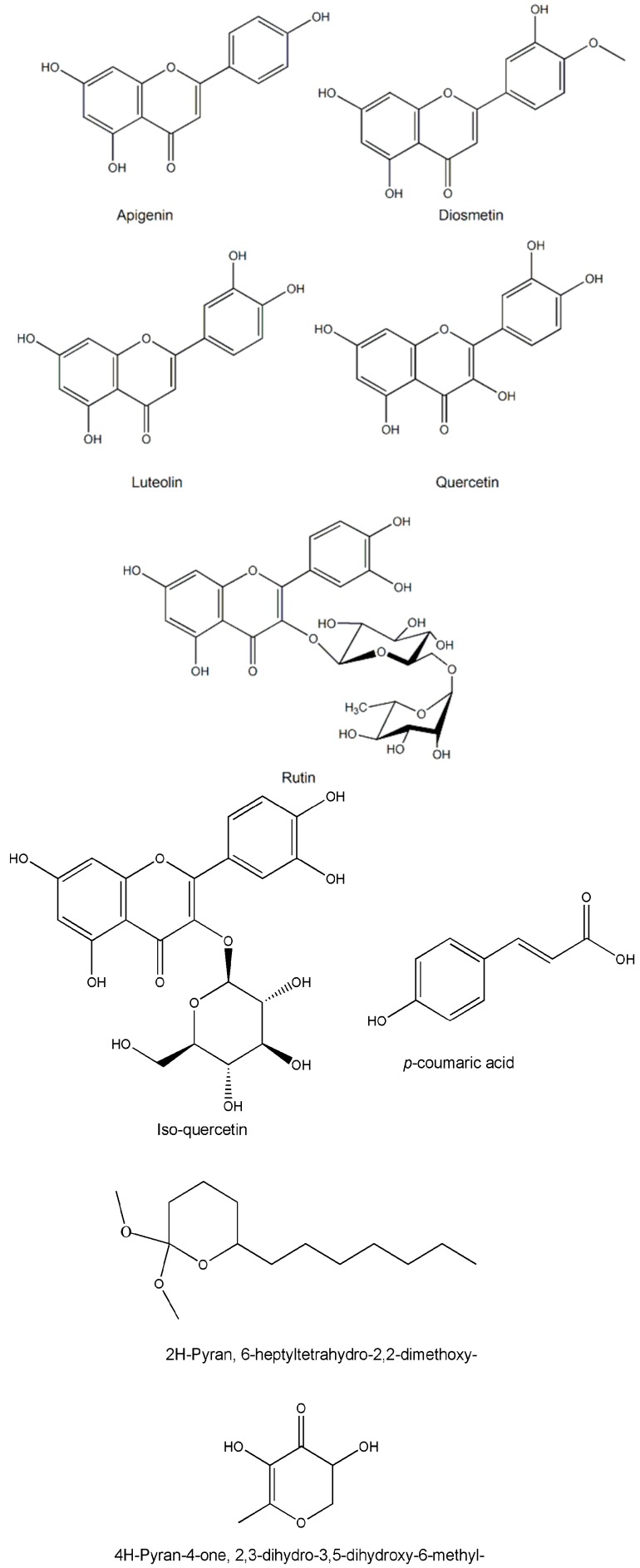
The structures of flavonoids and phenolic acids found in *L. reticulata*.

**Figure 3:**
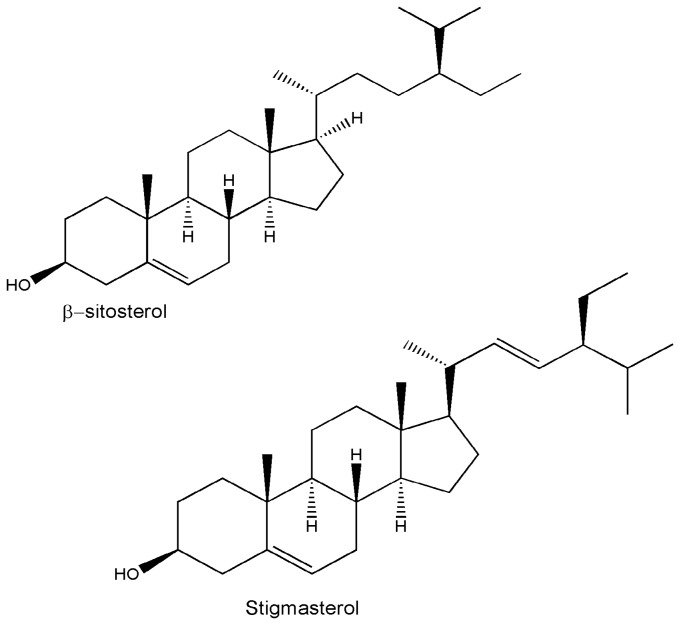
The structures of phytosterols found in *L. reticulata*.

**Figure 4:**
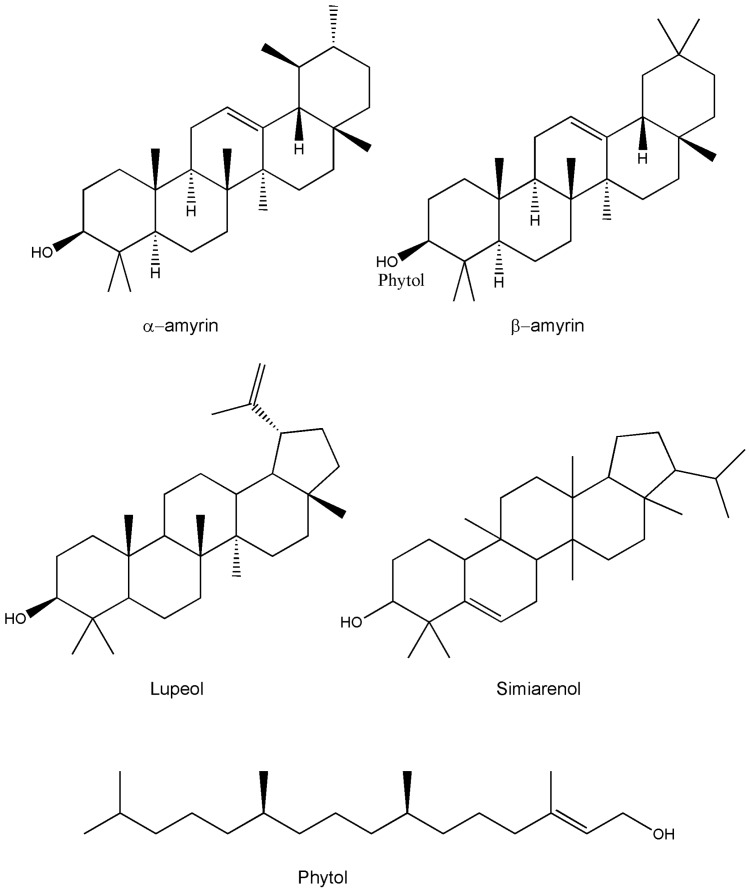
The structures of terpenes/terpenoids found in *L. reticulata*.

**Figure 5:**
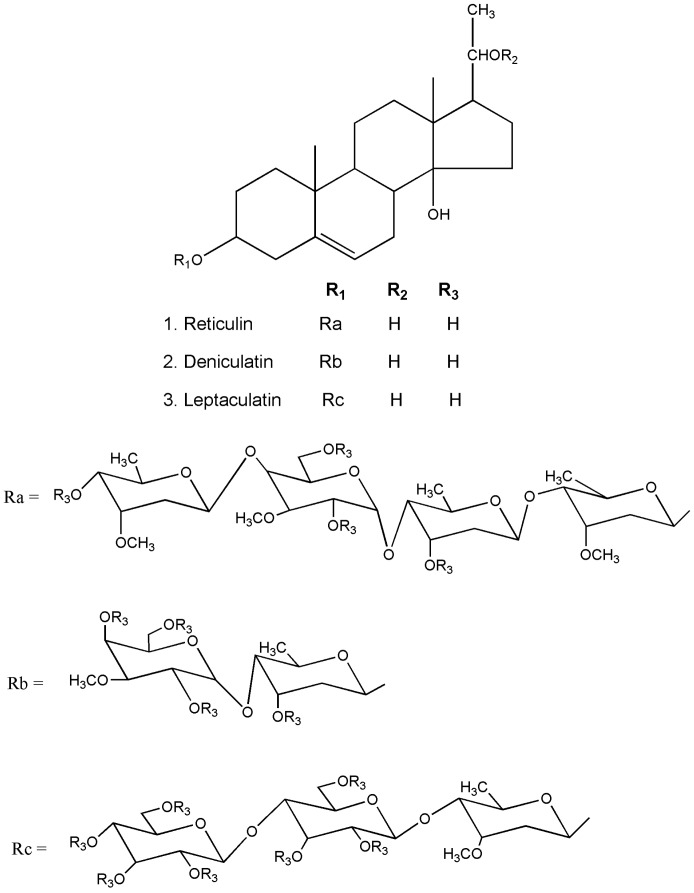
The structures of three novel pregnane glycosides found in *L. reticulata.*

**Figure 6:**
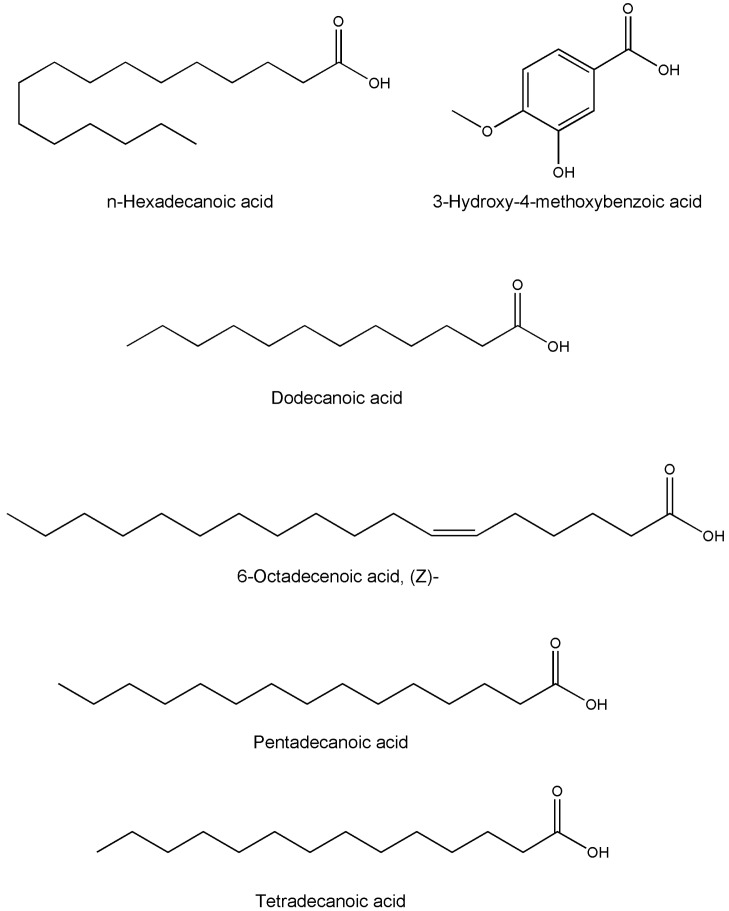
The structures of fatty acids found in *L. reticulata.*

**Figure 7:**
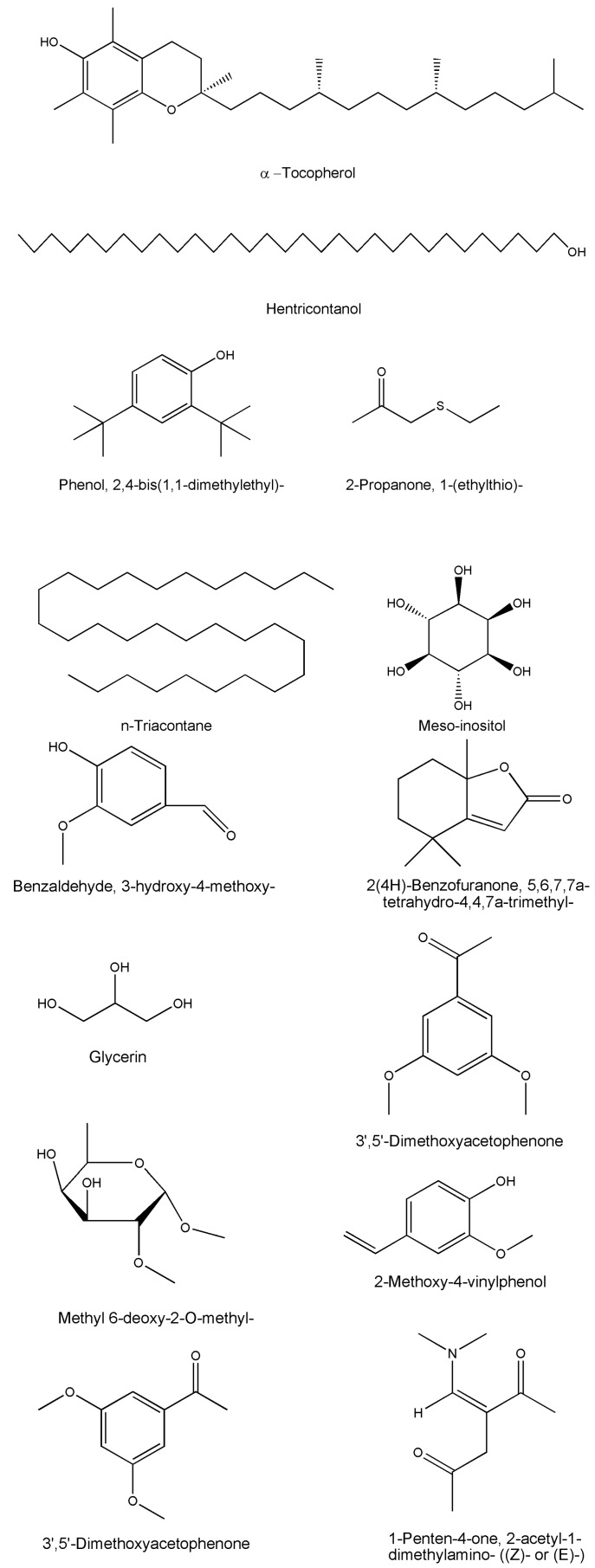

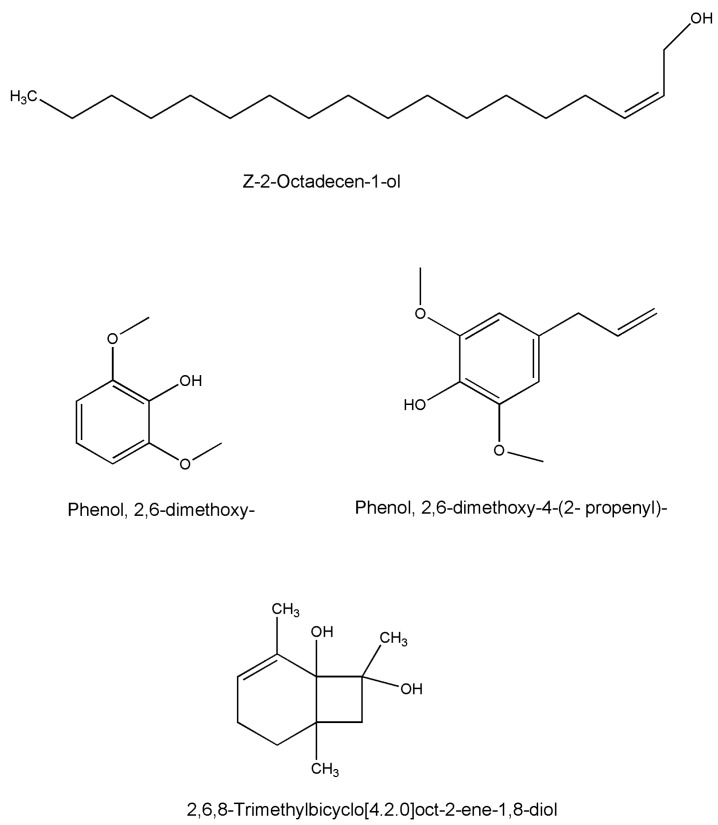
The structures of other few chemical constituents of *L. reticulata*.

Medicinal plants are recognzed as sources of a wide range of bioactive chemical entities. The enormous chemical diversity within each plant draws the attention of the pharma sector and has led to the development of many unique drugs. Our literature survey revealed a diverse chemical profile of *L. reticulata*, which included a total of 46 chemical compounds (Table 2). Structures of some of the major chemical components are depicted in Figure 2, Figure 3, Figure 4, Figure 5, Figure 6 and Figure 7.

In recent times, phytochemists and biologists are focusing on the isolation and identification of specific lead molecules of *L. reticulata* and understanding their therapeutic significance. The phytochemical composition and the content of bioactive compounds vary within the plant parts. Moreover, various factors such as geographical topographies, climatic condition, growing patters, and harvesting duration influence the accumulation pattern of biochemical constituents in plants [4,5,19,34]. In addition, the practice of extraction procedures can lead to discrepancies in the composition of plant compounds. To date, there are only a few studies related to the identification, isolation, and characterization of individual phytocompounds of *L*. *reticulata.* However, accurate documentation of well-characterized phytocompounds will benefit in proper understanding of their biological activities. In *L. reticulata*, quite a few classes of chemical compounds have been reported including terpenoids, phenolics, flavonoids, steroids, and esters. In this section, the phytochemical composition of *L. reticulata* is discussed in detail considering the above facts.

The preliminary qualitative tests have shown the occurrence of terpenoids, alkaloids, sterols, tannin, saponins, flavonoids, carbohydrates, and glycosides in the aerial parts of *L. reticulata* [35,36,37]. The *L. reticulata* leaves contain 6–7% of moisture, 17.5% of total nitrogen, 5.5 to 6.5% of total ash, 0.1% of insoluble ash, 0.6% of calcium, and 2.16 to 2.24% chlorides. Some of the other constituents identified are proteins, reducing sugars, gums, ketohexoses, pentoses, and volatile compounds [25,36]. A study by Hewageegana et al. [37], have stated that *L. reticulata* contains total ash (16.61%), water soluble ash (5.90%), acid insoluble ash (2.80%), dietary fiber (14.23%), protein (35.80%), carbohydrates (23.40%), crude fat (2.80%), iron (0.03%), magnesium (1.50%), and calcium (0.97%).

Likewise, carbohydrates, glycosides, flavonoids, tannins, saponins, phytosterols, free catechols, starches, and phenolic compounds were also reported in different solvent extracts of *L. reticulata* stems by researchers [38,39,40,41,42,43,44]. A flavone occurring as *C*-glycoside has also been identified in this plant [39]. In vitro grown cells of *L. reticulata* were shown to contain alkaloids, tannins, steroids, flavonoids, glycosides, terpenoids, and reducing sugars [40].

Krishna et al. [13], conducted the chromatographic separation of *L. reticulata* leaves and twigs solvent extracts. The infrared (IR) spectral data identified the presence of hentricontanol, α-amyrin, β-amyrin, and stigmasterol. Further, two flavones namely, diosmetin and luteolin, were also characterized for the first time by nuclear magnetic resonance (NMR) spectral analysis. Srivastav et al. [45], isolated three novel pregnane glycosides, namely reticulin (1), deniculatin (2), and leptaculatin (3) from the aerial parts of *L. reticulata.* Based on NMR, fast atom bombardment (FAB), and electron ionization mass spectral (EI-MS) data, the chemical structures of these compounds were elucidated as calogenin-3-*O*-β-cymaropyranosyl-(1→4)-*O*-3-*O*-methyl-α-d-galactopyranosyl-(1→4)-*O*-β-d-digitoxopyranosyl-(1→4)-*O*-β-d-cymaropyranoside (1), calogenin-3-*O*-3-*O*-methyl-α-d-galactopyranosyl-(1→4)-*O*-β-d-digitoxopyranoside (2), and calogenin-3-*O*-β-d-glucopyranosyl-(1→4)-*O*-β-d-glucopyranosyl-(1→4)-*O*-β-d-cymaropyranoside (3).

*L. reticulata* is found to be a rich source of many biologically active compounds such as triterpenoids, leptadenol, n-tricontane, cetyl alcohol, β-sitosterol, β-amyrin acetate, lupanol 3-O diglucoside, leptidin, luteolin, diosmetin, stigmasterol, and l-α-tocopherol. More than 23 commercial drugs are marketed using this plant as one of the key ingredients [11,25,44]. Hamrapurkar and Karishma [46] developed a high performance thin layer chromatography (HPTLC) method to identify stigmasterol and l-α-tocopherol acetate as two marker compounds in *L. reticulata*. They also determined the physicochemical properties, water-soluble and alcohol-soluble components, ash values, and moisture content in Jivanti. The leaves and twigs of the plant contain hentricontanol, α and β-amyrin, stigmasterol, γ-sitosterol, flavonoids, and luteolin. The fruits of this plant contain quercetin, isoquercetin, rutin, and hyperoside while the seeds contain meso-inositol, and its monomethyl ether [27].

TLC and HPTLC analysis revealed the occurrence of quercetin and isoquercetin in the dried aqueous extract of *L. reticulata* whole plant [36]. A simple HPTLC method was developed to identify alpha-amyrin, beta-sitosterol, lupeol, and n-triacontane from the methanolic extracts of *L. reticulata* whole plant [47]. Likewise, a simple and precise HPLC method was developed and validated to quantify *p*-coumaric acid in *L. reticulata* extract [43]. In another study, the presence of polyphenols such as rutin, quercetin and *p*-coumaric acid were identified in the ethyl acetate extract of *L. reticulata* leaves by using HPLC method [46]. Likewise, Prashanth et al. [47], developed and validated a sensitive and accurate HPTLC method to estimate rutin content in *L. reticulata* leaves. Using spectral (UV, IR, ^1^H-NMR, ^13^C-NMR and FAB-MS) studies, lupeol (a pentacyclic triterpenoid) was isolated from the chloroform extract of *L. reticulata* roots [48]. Gas chromatography-mass spectrometry (GC-MS) analysis of the ethanolic whole plant extract of *L*. *reticulata* revealed the presence of 31 phytocomponents as represented in Table 2 [49]. However, the occurrence of some reported chemical compounds such as 2,4-bis(1,1-dimethylethyl)phenol which is known to be a plastic additive, 1-(ethylthio)propan-2-one and 1,5,7-trimethylbicyclo[4.2.0]oct-4-ene-6,7-diol as native constituents of the plant appears doubtful.

Mammen et al. [28], studied the changes in chemical profile in *L. reticulata* collected in different seasons and different regions. Similar fingerprints were observed for the samples collected during summer, monsoon, and winter seasons. Differences in constituents among the extracts of samples collected from different places were quite negligible. The study on the geographical variations of *L. reticulata* revealed that the fingerprints of the three extracts of the plants collected from Gujarat, Maharashtra, and Kerala were very similar to each other. Thus, it was concluded that the environmental conditions did not affect the chemical constituents.

## 5. Pharmacological Properties of *L. reticulata*

*L. reticulata* is reported to possess a wide range of pharmacological activities and is used in many preparations for both human and veterinary usage (Table 3). Comprehensive information regarding the commercial preparations/formulations prepared by nutraceutical and pharmaceutical companies is presented in Table 4. Various biological activities of *L. reticulata* are discussed in the text. Also, some of the major commercially available *L. reticulata* containing herbal products and their health benefits are summarized in Table 4.

### 5.1. Antiabortifacient Effect

*L. reticulata* extract (Leptaden tablet) provides a good remedy for new mothers suffering from breast milk deficient or absence. This medicine has a galactagogue effect and also useful in the treatment of habitual abortions [50]. An experimental research in guinea pigs using radioimmunoassay suggested that Leptaden inhibits F2 alpha biosynthesis [51]. This helps in preventing abortion, since any increase in prostaglandins causes abortion or premature delivery. The effect of leptaden therapy is more beneficial over the combined treatment with progesterone. Also, it has been concluded that Leptaden therapy when done alone proved beneficial for the management of threatened abortion [52]. The researcher also described Leptaden as a non-hormonal and safe herbal drug. In case of threatened abortion, Leptaden can be used without hormonal treatment and requires no tests. It was found to be safe for both the mother and the child without any toxic side effects. leptaden can also be used to reduce uterine cramps of threatened abortion. This is probably because of the anti-prostaglandin effect of Leptaden. Leptaden tablets are also used for the treatment of uterine hemorrhages [53].

### 5.2. Antianaphylactic Activity

Padmalatha et al. [54], studied the effect of a polyherbal formulation of DLH-3041 (Himalaya Drug Company, Bengaluru, India), consisting of *L. reticulata* as one of the ingredient, on the active and passive anaphylaxis in rats using mesenteric mast cells and compared it with prednisolone and disodium cromoglycate. The herbal formulation (DLH-3041) showed significant protection against the mast cell degranulation in sensitized animals.

### 5.3. Antidepressant Effect

The effect of Malkanguni, a polyherbal formulation, in which *L. reticulata* is used for its antidepressant activity [55]. The drug was found to be effective without any side effects.

### 5.4. Antiepileptic Potential

Pushpa et al. [56], assessed the anti-epileptic potential of methanolic extract of *L. reticulata* against maximal electroshock (150 mA intensity for 0.2 s), pentylenetetrazol (70 mg/kg, i.p.), and lithium-pilocarpine-induced status epilepticus, respectively. It was found that the methanolic extract of *L. reticulata* showed significant effect against maximal electroshock and pentylenetetrazol but was not much effective against lithium-pilocarpine-induced status epilepticus and haloperidol-induced catalepsy.

### 5.5. Anti-Implantation Activity

The anti-implantation and hormonal (estrogenic) activities of ethanolic extract of *L. reticulata* were studied in albino rats by Rani et al. [57]. They concluded that *L. reticulata* possesses a significant estrogenic activity as well as a very strong anti-implantation activity.

### 5.6. Antimicrobial Activity

Vaghasiya and Chanda [58] studied the antibacterial activity of different solvent extracts of *L. reticulata* leaves against five Gram-positive, seven Gram-negative bacterial strains, and three fungal strains. They observed that acetone extract showed no activity against all the tested Gram-positive bacterial strains. However, it effectively inhibited two Gram-negative strains (*Klebsiella pneumoniae*, *Proteus mirabilis*, and *Citrobacter freundii*). While, the methanol extract was effective against both Gram-positive (*Staphylococcus aureus* and *S. epidermidis*) and Gram-negative strains (*Klebsiella pneumoniae* and *Proteus mirabilis*). Similarly, ethanol extracts of *L. reticulata* leaves exhibited potent antimicrobial activity against *Bacillus subtilis*, *Staphylococcus aureus*, *Escherichia coli*, *Pseudomonas aeruginosa*, *Klebsiella pneumoniae*, *Aspergillus flavus*, and *A. niger* [59]. In another study, different solvent extracts of the aerial parts of *L. reticulata* were reported to possess antimicrobial property. Among the extracts, chloroform and alcoholic extract inhibited *E. coli* and *P. aeruginosa* significantly, while petroleum ether extract was effective against *K. pneumoniae* [60]. The potential antibacterial actions of biosynthesized silver nanoparticles (AgNPs) in methanolic leaf extracts of *L. reticulata* were reported by Swamy et al. [61]. In this study, AgNPs were shown to effectively inhibit various pathogenic microbes such as *E. coli*, *K. pneumoniae*, *S. pneumoniae*, *M. luteus*, and *B. subtilis*. Similarly, Mishra et al. [62], indicated the potential benefits of the aerial parts of *L. reticulata* as a board-spectrum antifungal agent. Different solvent extracts were tested against *A. ruantii*, *A. flavus*, *Trichoderma viride*, *T. koningii*, *Candida tropicalis* and *C. albicans*. The minimum inhibitory concentration (MIC) of the different extracts against the tested strains was shown to be between 150 and 300 μg mL^−1^ concentrations. The methanol extract was effective in inhibiting all the tested fungal strains compared to other solvent extracts. The acetone and methanolic leaf extracts of *L. reticulata* exhibited antifungal activity against *C. tropicalis* and *C. tropicalis*, respectively [58]. The effect of ethanolic extract (50%) of the aerial part of *L. reticulata* was studied for its antifungal activity against *A. flavus* in rat animal model in vivo [63]. Visible healing and absence of the fungal hyphae were observed in the wound infected with *A. flavus.* From the experiment, it was found that the ethanolic extract of *L. reticulata* possesses antifungal activity against *A. flavus.*

### 5.7. Antitumor and In Vitro Cytotoxic Activity

Sathiyanarayanan et al. [64], evaluated the effect of leaf extract of *L. reticulata* against Dalton’s ascites lymphoma (DAL) in Swiss albino mice. They observed that the extract of *L. reticulata* had a significant inhibitory effect on the proliferationof tumor cell. In vitro study conducted by Mohanty et al. [65], showed that the ethyl acetate extract of naturally grown *L. reticulata* was effective in inhibiting MCF-7, HT-29, and L6 cells with IC_50_ values of 21 µg/mL, 26 µg/mL, and 22 µg/mL, respectively. The ethyl acetate extract of micropropagated *L. reticulata* exhibited cytotoxicity against MCF-7, HT-29, and L6 cells with IC_50_ values of 20 µg/mL, 30 µg/mL, and 18 µg/mL, respectively.

### 5.8. Antioxidant Activity

In vitro antioxidant study of methanolic extract of *L. reticulata* revealed a prominent free radical scavenging activity against diphenylpicrylhydrazyl (DPPH), hydroxyl, and nitric oxide radicals [66]. Antioxidant property of *L.reticulata* leaf extract was studied in rodents [67]. They found a significant increase in antioxidant enzymes, superoxide dismutase (SOD), and catalase (CAT), suggesting its antioxidant potential. Similarly, the DPPH free radical scavenging activity study showed the highest antioxidant potential in the ethyl acetate extract of *L. reticulata* with IC_50_ value of 267.13 µg/mL followed by the methanolic extract of *L. reticulata* with IC_50_ value of 510.15 µg/mL. Similarly, hydrogen peroxide scavenging and FeCl_3_ reducing method also recorded the highest antioxidant potential in ethyl acetate extract of *L. reticulata* with IC_50_ value of 234.1 µg/mL and 406.4 µg/mL, respectively [67]. In another study, antioxidant enzyme activity of in vitro regenerated and field-grown plants of *L. reticulata* were evaluated [68]. The results showed that CAT enzyme activity was observed in ex vivo grown plants, whereas, SOD and peroxidase enzyme activities were found to be highest in the acclimatized in vitro raised plantlets. The results of the study suggest the protective mechanism of this plant against various oxidative stresses.

### 5.9. Antipyretic, Analgesic, and Anti-Inflammatory Activity

Mohanty et al. [44], used formalin and λ–carrageenan-induced paw edema model followed by the determination of pro-inflammatory cytokines (IL-2, IL-6, and TNF-α) in the serum of adult Wister albino rats to evaluate the anti-inflammatory potential of whole plant solvent extracts of *L. reticulata*. The ethyl acetate extract at a dose of 600 mg kg^−1^ reduced edema by 60.59%, whereas, ethyl acetate fraction was effective enough to suppress edema by 59.24%. A significant reduction in the level of IL2, IL6, and TNF-α in the serum of animals treated with ethyl acetate extract (600 mg kg^−1^) indicated the potential anti-inflammatory activity of *L. reticulata*. The antipyretic and anti-inflammatory effects of the aqueous whole plantextract of *L. reticulata* were experimented in different animal models [69]. The results revealed that in all the animal models, a significant antipyretic and anti-inflammatory activity was observed at a dose of 200 mg kg^−1^ body weight and 400 mg kg^−1^ body weight, respectively. These findings suggested the possible use of aqueous extract of *L. reticulata* in the efficient management of inflammation and pyrexia in the future.

### 5.10. Antiulcer Activity

The aqueous leaf extract (100 mg kg^−1^ and 200 mg kg^−1^) of *L. reticulata* was evaluated for antiulcer activity in rats [70]. The results revealed a significant reduction in total acidity, acid volume, and ulcer index compared to control animals, suggesting the potential of *L. reticulata* leaves in the treatment of ulcers.

### 5.11. Anxiolytic Activity

Rajpurohit et al. [71], evaluated the anxiolytic activity in Wister albino rats by using elevated plus maze test, light-dark test, hole-board test, and social-interaction test models. The ethanolic leaf extract at a dose of 200 mg kg^−1^ and 400 mg kg^−1^ per body weight of the animal significantly showed potent anxiolytic activity compared to the control animals. The standard anxiolytic drug diazepam (2 mg kg^−1^) was used to compare the results.

### 5.12. Diuretic Activity

Treatment with whole plant extract of *L. reticulata* (aqueous and ethanolic) considerably improved the urine volume in normal rats compared to the control groups; however, the effect was relatively less compared to the standard (furosemide). The treatment also significantly increased the renal clearance of potassium, sodium, and chloride ions [72].

### 5.13. Galactagogue Property

Patel [73] for the first time drew attention by reporting the usefulness of Leptaden in preventing habitual abortion and later mentioned its lactogenic property. Later, the use of Leptaden tablet, a herbal formulation of *L. reticulata*, for the enhancement of milk yield in humans was practiced [74]. Likewise, clinical assessment showed the lactogenic property of Leptaden in the milk yield of dairy cows [75,76]. A clinical investigation showed that in most cases, Leptaden stimulated lactation in 12 h with easy flow, and lactation continued even after discontinuing the drugs [77,78]. The lactogenic property of *L. reticulata* and Leptaden tablets was studied on veterinary animals and reported a significant galactopoietic response in all cases [12]. The toxicity assessment of *L. reticulata* (aqueous extract) showed that rats safely tolerated up to a dose of 3.125 g/kg of Leptaden administered orally for three alternate days and three consecutive days [12]. For the first time, Dash et al. [79], studied the effect of Leptaden on milk production in Holstein and Brown Swiss breed of cows and buffaloes, determining the apparent effects of Leptaden on thyroid activity and the influence of the drug on the general health of cows. Anjaria et al. [80], conducted studies on stigmasterol and ether fraction isolated from *L. reticulata* for lactogenic effect on rats. It was found that both the active ingredients had lactogenic effect. Baig et al. [81], assessed the effect of polynutrient formulation Galactin Vet (The Himalaya Drug Company) with *L. reticulata* as one of the ingredient for increase in average milk production in cattle. It was found that Galactin Vet improved milk yield and fat percentage in dairy cows with no adverse effects on the health of the animals, suggesting its safe use as a galactagogue in dairy animals.

### 5.14. Hepatoprotective Activity

Nema et al. [38], investigated the hepatoprotective activity of the stems extracts of *L. reticulata* on paracetamol-induced hepatic damage in albino rats. The hepatoprotective action of ethanolic extract of *L. reticulata* was evidenced by a significant reduction in the elevated serum glutamic oxaloacetic transaminase, serum glutamic pyruvic transaminase, and alkaline phosphatase level. The ethanolic extract of *L. reticulata* showed significant hepatoprotective activity, and the efficacy of the extract was almost comparable to that of the standard drug LIV-52.

### 5.15. Immunomodulatory Activity

In a study by Girishkumar et al. [82], whole plant aqueous extract of *L. reticulata* was reported to offer superior protection against immunosuppression induced by chromate (VI). The results confirmed the possibilities of using *L. reticulata* for modulating and alleviating the chromate (VI)-induced immunosuppression. Likewise, the immunomodulatory potential and antioxidant activities of ethanolic leaf extract of *L. reticulata* was evaluated by Pravansha et al. [67]. The study showed that *L. reticulata* extract (100 and 200 mg/kg) significantly induced a delayed type of hypersensitivity reaction, increased antibody titer values in a dose-dependent manner, increased neutrophil adhesion (%) to nylon fibers, and the rate of phagocytosis. In addition, there was a significant increase in hematological profile, reduced glutathione, superoxide dismutase, and catalase activities. This demonstrates the potential immunomodulatory and antioxidant properties of *L. reticulata*.

### 5.16. Treatment of Oligospermia

Speman tablets (Himalaya Drug Co.) possess oligospermic effects [83]. Increase in sperm count and improved motility was noticed. Speman was found to be effective in the treatment of oligospermia without any side effects.

### 5.17. Other Studies

Agarwal et al. [84], reported that the aqueous extract of *L. reticulata* has revealed that the drug possesses a potent and prolonged hypotensive action in anaesthetized dog. In addition, there was no acute or chronic toxicity observed in rats. The leaves and roots are used in asthma, cough, and against skin infections such as ringworms and wounds [85]. Marya et al. [86], reported that when Speman tablet 325 mg (Himalaya Drug Co., Bombay, India) was used in the treatment of benign prostatic hyperplasia, the drug brought symptomatic relief by improving dynamics of micturition. Wakade et al. [66], observed the cardioprotective effect of *L.* reticulata against myocardial oxidative damages induced by adriamycin in rat models. The ethyl acetate fraction and methanol fraction of *L. reticulata* were shown to exhibit a dose-dependent lipid peroxidation inhibition activity. The highest inhibitory activity (71.2%) was observed in ethyl acetate fraction followed by methanol fraction with 58.18% [65].

## 6. Biotechnological Aspects of *L. reticulata*

Plant tissue culture as a biotechnological approach, is widely employed as an alternative source to obtain sufficient genuine planting materials for commercial cultivation. In addition, many endangered medicinal plant species can be conserved [87,88,89,90,91] and there is a possibility of an increased production of plant secondary metabolites useful in pharmaceuticals, cosmeceutical, and food industries [18]. A specific strategy is required to produce active principles from in vitro cultured cells [18,92]. Some of the factors that influence the in vitro culture includes type of the explants, media composition, type of plant growth regulators, different growth conditions (temperature, light sources, and humidity), types of cultures (solid cultures and agitated liquid cultures), cell line section, and the use of elicitation technology [18,90,93]. In vitro plant regeneration can be obtained through direct organogenesis or indirect organogenesis, which involves callus interphase [87,89,90]. The established in vitro culture system will be very useful for further genetic manipulation studies or large-scale secondary metabolites production. The expanding interest in the therapeutic potential of *L. reticulata* around the globe has resulted in several biotechnological research activities. The following section highlights the most important research reports on the biotechnological aspects of *L. reticulata*.

### Plant Tissue Culture, Plant Regeneration and Conservation Studies

Because of well-defined pharmacopeia, growing urbanization, indiscriminate collection, and overexploitation of natural resources, many of the important medicinal plants are facing extinction. In order to cope up with the alarming situation, many biotechnological tools provide a valuable alternative for plant diversity studies, management of genetic resources, and conservation. One such important tool is plant tissue culture technology, which has a significant role in producing true to type, rapid, and mass production of disease-free plants under controlled conditions [18]. The micropropagation technology for mass multiplication has been tried out for of this species as well. So far, very limited number of reports is available on micropropagation through direct organogenesis. Arya et al. [94], reported that there is an urgent need for developing a non-conventional approach for mass propagation, conservation, and sustainable utilization of *L. reticulata*. Three to four shoots were developed from a single node using MS medium along with (25 mg/L each of adenine sulfate, arginine, citric acid, 50 mg/L ascorbic acid) containing 0.6 µM indole-3-acetic acid (IAA) and 9 µM benzyladenine (BA). Shoots are further multiplied by sub-culturing on to fresh medium containing 0.6 µM IAA and 2.2 µM BA. Individual shoots were rooted ex vitro after treating with 123 µM of indole-3-butyric acid (IBA) and β-naphthoxyacetic acid. The rooted plants were transferred to a net pot containing sterile soilrite. The hardened plants were transferred to polybags after 15 days and then transferred to the field. Somatic embryogenesis and regeneration of plants using leaf explants of *L. reticulata* was reported by Hariharan et al. [95]. Embryogenic callus was initiated and established successfully on MS medium supplemented with 6-BA (2.0 mg/L) and α-naphthaleneacetic acid (NAA) (0.5 mg/L). The embryoids developed were sub cultured on to hormone-free MS medium or on medium with lower concentration of hormones. The embryoids were later germinated on MS medium supplemented with 1.0 mg/L kinetin (Kn). The plants thus regenerated were transferred to the field for hardening, and 50% survival was achieved. In another study, BA-induced somatic embryogenesis and plant regeneration from different explants of *L. reticulata* was explained [96]. Among the different explants used, shoot tip and nodal segment are found to be morphologically active on MS medium supplemented with 8.87 µM BA and 2.46 µM IBA and successfully included embryogenic callus. The embryogenic calli were transferred to a suspension culture to facilitate the growth of the embryo. MS medium (1/2 strength) along with 1.44 μM gibberellic acid (GA_3_) and BA (0.22 or 0.44 μM) was found to be most efficient in the conversion of embryo to plantlets. Plants were transferred to net pots and subsequently to the field with 80% survival rate [96]. Likewise, plant regeneration through somatic embryogenesis was also achieved by using stem explants of *L. reticulata* [19]. MS medium supplemented with 3% sucrose, 2.68 μM NAA, and 2 μM BAP was proved to be the best medium for callus induction. MS liquid medium was superior to solid medium for cell proliferation. The developed shoots were subjected to rooting on half strength MS medium with 4.90 μM IBA and established in the field with 75% survival rate. Multiple shoots were induced on MS medium containing 5.0 mg/L 6 BA [19]. The plants were multiplied using 1.5 mg/L BA and 0.5 mg/L kinetin (Kn). Well-grown shoots were transferred to MS medium along with 200 mg/L IBA for rooting. Studied have shown that plant growth regulators significantly influence in vitro morphogenesis of *L. reticulata* [97,98]. The MS basal medium supplemented with IBA (1 mg/L) and Kn (10 mg/L) induced multiple shoots as well as callus at the base of nodal explants. Later, callus was subjected to organogenesis using NAA (1.5 mg/L), Kn (10 mg/L) or IBA (1 and 1.5 mg/L) with Kn (2 mg/L) [98]. While, Rathore et al. [99] reported MS medium containing 5.0 mg/L of BA and ammonium sulfate was effective in shoot multiplication. In another study, the best response for shoot multiplication was obtained on MS media supplemented with 0.25 mg/L BA and 0.25 mg/L Kn. Full strength MS media containing 2 mg/L IBA induced maximum rooting response. Interestingly, the use of 200 mg/L activated charcoal in in MS media was equally effective in inducing roots [90]. The effect of various carbon sources and natural additives on in vitro morphogenesis of *L. reticulata* was studied by Sudipta et al. [90]. According to them, 2% sucrose followed by 2% table sugar effectively influenced the shoot multiplication rate as well as plant physiology. Among natural additives, 10% coconut water was found to be the best for inducing the highest number of multiple shoots. In addition, the cost of culture media was reduced by using tap water and table sugar in place of sucrose and distilled water. The use of picloram (2 mg/L) was effective in forming friable callus from leaf explants [40]. Further, phytochemical screening study indicated both exogenous and endogenous production of secondary metabolites in suspension cultures. The presence of alkaloids, glycosides, saponins, flavonoids, terpenoids, reducing sugars and tannins was noticed in both spent media and cells. While, steroids were produced endogenously and absent in the media.

## 7. Current Demand

The escalating demand for medicinal plants in India for domestic, national, and international market has increased in recent times. According to the World Health Organization (WHO), the demand is growing at the rate of 15 to 25% annually. Currently, the global market for herbal medicinal products is approximately USD 62 billion. It is speculated that this demand will grow up to USD 5 trillion by the year 2050 [3,6]. Demand for some important medicinal plants has increased to an extent that it has resulted in the rapid loss of biodiversity as well as the extinction of such important species. In recent years, the demand for medicinal and aromatic plants has grown rapidly because of accelerated local, national, and international interest notably from the pharmaceutical, nutraceutical and aroma industries [1,2,9,18]. *L. reticulata* is of great demand in both local and international market, with dry powder costing INR 211/kg and flowers up to INR 80/kg [36,97]. In India, the current commercial market price of 100% natural *L. reticulata* powder is about USD 8 [100,101,102]. Likewise, another commercial supplier has quoted the approximate price of INR 100/kg of *L. reticulata* raw material [101]. It was mentioned that the natural and current practices of propagation are unable to meet the huge demand for this plant, and the species is currently threatened in nature [96]. In a study, it has been mentioned that the huge demand and the multipurpose usage of this plant by pharma industries were the reasons behind the current endangered status of this plant [30]. As per another report, the market price of *L. reticulata* dry roots was at INR 65/kg, and market demand was at 23 tons/year in 2006–2007 [101,102].

## 8. Adulteration and Authentication of *L. reticulata*

Because of the limited cultivation base, the scarcity of planting materials and the global demand for *L. reticulata* herbal materials have drastically increased in recent times. Thus, traders are certainly prompted to introduce various substitutes and adulterants for *L. reticulata* dry herb in the market. The authentication of quality materials is essential in order to sustain the herbal industries and for patients to benefit maximum from the herbal products [18]. To date, there are no standard protocols to identify and differentiate *L. reticulata* from other possible powdered herbal raw materials. It is very difficult to segregate on the basis of morphology or anatomical properties as *Leptadenia* genus closely resembles other related species. Hence, it is often confused with other plant species and thus prone to adulteration in the Indian market by other cheap plant powdered materials. A sensitive HPTLC method was to estimate the content of rutin, stigmasterol, and dl-α-tocopherol acetate in *L. reticulata* leaves [48,57]. They have suggested that it can be used as a marker for identifying *L. reticulata* precisely for quality analysis regularly. Raval and Mishra [103] designed a standard procedure based on the physico-chemical and chromatographic approaches in order to differentiate *L. reticulata* samples from other substitutes or adulterants. They used HPTLC to detect β-carotene from the aerial parts of the plant extracts. Likewise, Mammen et al. [22], conducted the biomarker study to distinguish *L. reticulata* from another morphologically similar plant *Ichnocarpus frutescens* to detect adulteration. Likewise, a HPTLC method to validate the occurrence of four compounds, namely alpha-amyrin, beta-sitosterol, lupeol, and n-triacontane from *L. reticulata* [28]. This simple method can benefit the regular process of quality control of *L. reticulata* herbs. The dried aqueous extract of *L. reticulata* (whole plant) subjected to several physico-chemical analyses including thin layer chromatography and HPTLC revealed the presence of flavonoid glycosides quercetin and isoquercetin [36]. More recently, Girija and Shree [104] developed a simple quality control method to differentiate the raw drug *L. reticulata* from other raw herb materials based on their histological, histochemical, and phytochemical properties.

## 9. Conclusions and Future Scope

This review documents the available scientific data on the botanical, propagational, phytochemical, biological, and conservational aspects of *L. reticulata*. This multi-utilitarian medicinal herb offers several promising medicinal values, and hence, can be used in the present day therapeutic practices to treat various human ailments. *L. reticulata* with its revitalizing, rejuvenating, and lactogenic properties can be used as the main component in many herbal formulations. This herb contains several classes of bioactive compounds. The agronomic practices, including vegetative propagation, crop management, and harvesting conditions, are well documented to support its improved large-scale cultivation in the field. Some of the scientific practices such as selection of elite planting material, well-timed planting, timely application of soil nutrients, appropriate use of growth regulators, irrigation, weed elimination, and harvesting stage are essential for sustainable cultivation and production. Furthermore, the utilization of biotechnological tools such as micropropagation, molecular markers, and cell culture are also emphasized in order to validate these methods in future applications. The compiled tissue culture data will certainly pave a way toward developing a low-cost tissue culture methodology for propagating elite germplasm of *L. reticulata* through micropropagation. At present, *L. reticulata* is a threatened endangered plant because of its overexploitation, unscientific harvesting, and habitat loss. Therefore, future research should focus on its conservational aspects. Application of novel techniques such as cell culture, genetic engineering, and utilization of bioreactors will also supplement the in vitro production of bioactive compounds from *L. reticulata*. Although the biological properties of *L. reticulata* are well understood, most studies were restricted to crude extracts and few pure isolated compounds. Moreover, many biologically active compounds are yet to be identified. Most of the phytochemicals described as constituents of the title plant are rather widespread in plants and not particularly characteristic for *L. retiulata*. It remains to be shown in further detailed studies which of them, or which of their combinations, are to be held responsible for the many described biological activities. Therefore, considerable research is required in the field of plant identification, extraction, isolation, and characterization of other biologically active compounds from this herb so as to fully claim its traditional uses into evidence-based human use. Additional in vitro and in vivo studies should also be considered to evaluate and understand in detail the possible underlying molecular mechanisms of action of each pure compound of *L. reticulata.* Further toxicity studies and clinical trials must be encouraged to ensure safety of this medicinal plant for human use. There is a need to establish quality control methods to avoid possible misidentification or adulteration of *L. reticulata* with other plant materials. Overall, this review highlights the therapeutic potential of *L. reticulata*, considering the current market demand, and promotes future research on its conservation and large-scale cultivation by adopting proper agronomical practices.

## Figures and Tables

**Table 1:** Vernacular names/Synonyms of *Leptadenia reticulata*.

Language	Vernacular Names (Language)
Hindi	*Dori*
Bengali	*Bhadjivai*
English	*Jiwanti or Jeevanti*
Gujarati	*Methidodi*, *Dodi saka/Dodi Saag*, *Dori*
Marathi	*Haranvel*, *Hiranvel*
Kannada	*Hiriyahalle*
Sanskrit	*Madhusrava*, *Jivniya*, *Jivapushpa or Jivani*
Tamil	*Palaikkodi*
Telugu	*Kalasa*

**Table 2:** The known volatile constituents of *L. reticulata.*

Compound Name	Formula	Plant Part	Method	References
**Flavonoids**				
Apigenin	C_48_H_80_O_17_	Stem bark	-	[14,15]
Diosmetin	C_16_H_12_O_6_	Whole plant/leaves/twigs	Chromatography/HPTLC/NMR	[14,15]
Luteolin	C_16_H_12_O_6_	Whole plant/leaves/twigs	Chromatography/HPTLC/NMR	[14,15]
Rutin	C_27_H_30_O_16_	Leaves	HPLC/HPLC	[27,43,46,47]
Quercetin	C_15_H_10_O_7_	Whole plant	TLC/HPTLC	[36,44]
Iso-quercetin	C_21_H_20_O_12_	Whole plant	TLC/HPTLC	[12,29]
4*H*-Pyran-4-one, 2,3-dihydro-3,5-dihydroxy-6-methyl-	C_6_H_8_O_4_	Whole plant	GC-MS	[49]
2*H*-Pyran, 6-heptyltetrahydro-2,2-dimethoxy-	C_14_H_28_O_3_	Whole plant	GC-MS	[49]
**Phenolic compound**				
*p*-coumaric acid	C_9_H_8_O_3_	Whole plant/leaves	HPLC	[29,40,43,44]
**Terpenes/Terpenoids**				
α-amyrin	C_30_H_50_O	Whole plant/leaves/twigs	Chromatography/HPTLC/IR	[13,15,28,47]
β-amyrin	C_30_H_50_O	Whole plant/leaves/twigs	Chromatography/HPTLC/IR	[11,25,27,44]
Lupeol	C_30_H_50_O	Whole plant/roots	HPTLC/NMR	[42,48]
Simiarenol	C_30_H_50_O	Whole plant	-	[14]
Phytol	C_20_H_40_	Whole plant	GC-MS	[49]
**Esters**				
Benzene carboxylic acid	C_7_H_6_O_2_	Whole plant	GC-MS	[49]
Ethyl hydrogen succinate	C_6_H_10_O_4_	Whole plant	GC-MS	[49]
Hexadecanoic acid, ethyl ester	C_18_H_36_O_2_	Whole plant	GC-MS	[49]
4-Oxazolecarboxylic acid, 4,5-dihydro-2-phenyl-, 1-methylethyl ester	C_13_H_15_NO_3_	Whole plant	GC-MS	[49]
9,12-Octadecadienoic acid, ethyl Ester	C_20_H_36_O_2_	Whole plant	GC-MS	[49]
9-Octadecenoic acid, ethylester	C_20_H_38_O_2_	Whole plant	GC-MS	[49]
**Fatty acids**				
Dodecanoic acid	C_12_H_24_O_2_	Whole plant	GC-MS	[49]
*n*-Hexadecanoic acid	C_16_H_32_O_2_	Whole plant	GC-MS	[49]
3-Hydroxy-4-methoxybenzoic acid	C_8_H_8_O_4_	Whole plant	GC-MS	[49]
6-Octadecenoic acid, (*Z*)-	C_18_H_34_O_2_	Whole plant	GC-MS	[49]
Pentadecanoic acid	C_15_H_30_O_2_	Whole plant	GC-MS	[49]
Tetradecanoic acid	C_14_H_28_O_2_	Whole plant	GC-MS	[49]
**Pregnane glycosides**				
Deniculatin	C_34_H_56_O_11_	Aerial parts	NMR/FAB/EI-MS	[45]
Leptaculatin	C_40_H_66_O_16_	Aerial parts	NMR/FAB/EI-MS	[14,15]
Reticulin	C_34_H_56_O_11_	Aerial parts	NMR/FAB/EI-MS	[15,16,29]
**Steroids**				
β-sitosterol	C_29_H_50_O	Whole plant/leaves/twigs	TLC/HPTLC	[14,15,44,46]
Stigmasterol	C_29_H_48_O	Whole plant/leaves/twigs/roots	Chromatography/HPTLC/IR	[12,14,15,25,29,44,46]
**Others (Carbohydrates, carboxylic acid, ethers, alcohols, aldehydes etc.)**				
Benzaldehyde, 3-hydroxy-4-methoxy-	C_8_H_8_O_3_	Whole plant	GC-MS	[49]
2(4*H*)-Benzofuranone, 5,6,7,7a-tetrahydro-4,4,7a-trimethyl-	C_11_H_16_O_2_	Whole plant	GC-MS	[50]
Glycerin	C_3_H_8_O_3_	Whole plant	GC-MS	[49]
3′,5′-Dimethoxyacetophenone	C_10_H_12_O_3_	Whole plant	GC-MS	[49]
Hentricontanol	C_31_H_64_O	Leaves/twigs	Chromatography/HPTLC/NMR	[14,15]
Methyl 6-deoxy-2-*O*-methyl-	C_8_H_16_O_5_	Whole plant	GC-MS	[49]
2-Methoxy-4-vinylphenol	C_9_H_10_O_2_	Whole plant	GC-MS	[49]
Meso-inositol	C_6_H_12_O_6_	Whole plant	-	[12]
*Z*-2-Octadecen-1-ol	C_18_H_36_O	Whole plant	GC-MS	[49]
1-Penten-4-one, 2-acetyl-1-dimethylamino- ((*Z*)- or (*E*)-)	C_9_H_15_NO_2_	Whole plant	GC-MS	[49]
Phenol, 2,6-dimethoxy-	C_8_H_10_O_3_	Whole plant	GC-MS	[49]
Phenol, 2,6-dimethoxy-4-(2-propenyl)-	C_11_H_14_O_3_	Whole plant	GC-MS	[49]
Phenol, 2,4-bis(1,1-dimethylethyl)-	C_14_H_22_O	Whole plant	GC-MS	[49]
2-Propanone, 1-(ethylthio)-	C_5_H_10_O	Whole plant	GC-MS	[49]
α-tocopherol	C_29_H_50_O_2_	Roots/stems/leaves	HPTLC	[12,13,25,44,46]
2,6,8-Trimethylbicyclo[4.2.0]oct-2-ene-1,8-diol	C_11_H_18_O_2_	Whole plant	GC-MS	[49]
*n*-triacontane	C_30_H_62_	Whole plant	HPTLC	[45]

**Table 3:** Pharmacological properties of *L. reticulata* different parts.

Pharmacological Activity or Therapeutic Use	Part of the Plant Used or Extract	Animal Used	References
Antiabortifacient effect	Plant powder and Leptaden tablet	Human, Guinea pig	[50,51,52,53]
Anti-anaphylactic activity	The herbal formulation (DLH-3041)	Normal rats	[54]
Antidepressant effect	A polyherbal formulation (Malkanguni)	Human	[55]
Anti-epileptic potential	Leaf	-	[56]
Anti-implantation activity	Leaf	Albino rats	[57]
Antimicrobial activity	Whole plant, stem, leaf and root	-	[58,59,60,61,62,63]
Antitumor/in vitro cytotoxic activity	Whole plant, leaf, in vitro plants, callus	Swiss albino mice	[64,65]
Antioxidant activity	Leaf, in vitro plants, callus, whole plant	Normal rats	[66,67,68]
Antipyretic, analgesic, and anti-inflammatory activity	Whole plant	Albino rats	[44,69]
Antiulcer activity	Leaf	Normal rats	[70,71]
Anxiolytic activity	Leaf	Wister albino rats	[72]
Cardioprotective activity	Leaf	Normal rats	[66]
Diuretic activity	Whole plants	Normal rats	[72]
Galactogogue property/Lactogenic effect/improving milk quality	Whole plant and Leptaden (Herbal formulation)	Human, cows, normal rat	[12,73,74,75,76,77,78,79,80,81]
Hepatoprotective activity	Stem	Swiss albino mice	[38]
Immunomodulatory activity	Whole plant, leaf	Swiss albino mice	[67,82]
Rejuvenating properties	Whole plant	Humans	[83,84,85,86]
Treatment of oligospermia	The Speman tablet (Himalaya Drug Co., Bengaluru)	Human	[83]
Treatment of asthma, bronchitis and throat trouble	Whole plant	Human	[11]
Treatment of Skin infection against ringworms and wounds	Whole plant	Human	[85]
Treatment of oligospermia	Speman (Herbal formulation)	Human	[86]
Treatment of benign prostatic hyperplasia, Prostatitis	Speman (Herbal formulation)	Human	[86]
Vasodilator, Hypotensive effect	Stem	Dog	[84]

**Table 4:** List of some of commercial products that contain *L. reticulata* as one of the major ingredient and their potential health benefits.

Name of the Product	Company	Uses
Confido (Speman forte)	Himalaya Drug House, Bengaluru, India	Useful in oligospermia
Speman	Himalaya Drug House, Bengaluru, India	Helps in Spermatogenesis
Galactin Vet (bolus)	Himalaya Drug House, Bengaluru, India	Stimulate activity of alveolar tissue, stimulate lactogenesis, improve fat percentage
Speman forte vet	Himalaya Drug House, Bengaluru, India	Spermatogenic and increases libido
Speman vet	Himalaya Drug House, Bengaluru, India	Promotes spermatogenesis
Himalaya^TM^ Chyavanprasha	Himalaya Drug House, Bengaluru, India	Useful in debilitating disorders like cough, cold, infection. Boost immunity of the body
Calshakti	Intas Pharmaceuticals Ltd., Ahmedabad, India	Feed suppliments (Animal health)
Safe herbs	VASU Healthcare Pvt. Ltd., Hyderabad, India	Improves lactation (Women care)
Jivanti Powder/capsule	Evaidyaji Wellness Pvt. Ltd., Jaipur, India	Used in allergic response, constipation, cardiac and bleeding disorders, possess diuretic property
Enviro Care	Satveda, India/Herbs Forever, Los Angeles, CA, USA	Used as antioxidants, immunobuilder, rejuvenating and vitalizing tonic
Antisept	Zydus Cadila Healthcare Ltd., Ahmedabad, India	Possess antiseptic and antibacterial properties
Praas™ (Chyawanprash)	Komal Herbals, Inc., Sewickley, PA, USA	Enhance general health, increase mental and physical energy, increase resistance to disease

## Abbreviations

The following abbreviations are used in this manuscript:

AgNPs	Silver nanoparticles
AMF	Arbuscular mycorrhizal fungi
BA	Benzyladenine
CAT	Catalase
DAL	Dalton’s ascites lymphoma
DPPH	Diphenylpicrylhydrazyl
EI-MS	Electron ionization mass spectrum
FYM	Farm yard manure
FAB	Fast atom bombardment
GA3	Gibberellic acid
GC-MS	Gas chromatography-mass spectroscopy
HPLC	High pressure liquid chromatography
HPTLC	High performance thin layer chromatography
IAA	Indole-3-acetic acid
IBA	Indole-3-butyric acid
IR	Infrared
Kn	Kinetin
MIC	Minimum inhibitory concentration
NAA	α-naphthaleneacetic acid
NMR	Nuclear magnetic resonance
NPK	Nitrogen, Phosphorus, Potassium
SOD	Superoxide dismutase
TLC	Thin layer chromatography
